# Using digital pathology to standardize and automate histological evaluations of environmental samples

**DOI:** 10.1093/etojnl/vgae038

**Published:** 2025-01-06

**Authors:** Philip Tanabe, Daniel Schlenk, Kristy L Forsgren, Daniela M Pampanin

**Affiliations:** National Ocean Service, National Centers for Coastal Ocean Science, National Oceanic and Atmospheric Administration, Charleston, SC, United States; Department of Environmental Sciences, University of California, Riverside, Riverside, CA, United States; Department of Biological Science, California State University, Fullerton, Fullerton, CA, United States; Department of Chemistry, Bioscience and Environmental Engineering, University of Stavanger, Stavanger, Norway

**Keywords:** histopathology, digital pathology, automation, machine learning, environmental monitoring

## Abstract

Histological evaluations of tissues are commonly used in environmental monitoring studies to assess the health and fitness status of populations or even whole ecosystems. Although traditional histology can be cost-effective, there is a shortage of proficient histopathologists and results can often be subjective between operators, leading to variance. Digital pathology is a powerful diagnostic tool that has already significantly transformed research in human health but has rarely been applied to environmental studies. Digital analyses of whole slide images introduce possibilities of highly standardized histopathological evaluations, as well as the use of artificial intelligence for novel analyses. Furthermore, incorporation of digital pathology into environmental monitoring studies using standardized bioindicator species or groups such as bivalves and fish can greatly improve the accuracy, reproducibility, and efficiency of the studies. This review aims to introduce readers to digital pathology and how it can be applied to environmental studies. This includes guidelines for sample preparation, potential sources of error, and comparisons to traditional histopathological analyses.

## Graphical abstract

**Figure:**
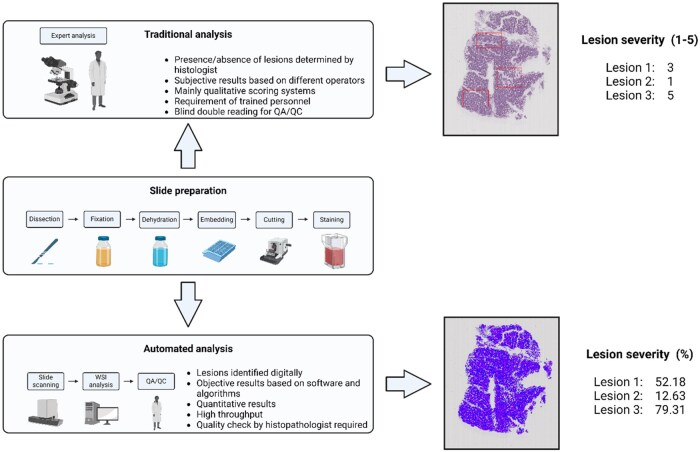

## Introduction

Histopathology is widely used as a biological endpoint in environmental monitoring studies investigating the biological effects of contaminants (Au, 2004; Beyer et al., 2017; Howard, 2004; International Council for the Exploration of the Sea, 2022; Lent et al., 2021; Wang et al., 2019; Yancheva et al., 2016; Zuykov et al., 2013). Assessment of lesions and tissue alterations can determine the health and fitness status of individuals, from which the general condition of populations or even whole ecosystems can be drawn. Histopathological evaluations of tissues can also be used to detect early signs of disease and predict harmful effects (Cruz-Flores & Cáceres-Martínez, 2020). For example, histological lesions and tissue alterations detected in gonads of aquatic organisms may indicate potential impairment of their reproductive capacity, whereas abnormalities in gills and digestive glands (e.g., in bivalve mollusks) can indicate harmful effects that may compromise survival and fitness of affected individuals (Binelli et al., 2004).

Although improvements in molecular biology techniques and reduced costs in omics analyses have allowed sublethal effects to be increasingly measured at molecular levels, they will likely not replace histopathological evaluations. Molecular biomarkers can provide evidence of exposure to contaminants and inform about mechanisms of action but do not always translate to physiologically relevant effects. For example, the induction of vitellogenin in male fish does not always correlate with population impacts (Mills et al., 2003; Seki et al., 2002). Histopathological evaluations look at higher levels of biological organization, such as tissues and organs, which can reveal abnormalities that are more likely to be physiologically relevant, such as formation of ovotestis (Seki et al., 2002). This highlights the need to include histopathological assessments in environmental surveys, because they can inform us of the general health of aquatic environments as well as drive the establishment and revision of guidelines for environmental preservation.

Although histological lesions and tissue alterations provide significant information, there is a deficit of experienced histopathologists relative to the number of studies utilizing histopathological evaluations. Wolf and Maack (2017) reviewed 189 studies to draw attention to challenges that can affect histopathology results and found 46% of evaluated studies to be equivocal, dubious, or to have no credibility. Several of the studies that fell into these categories were suspected to have utilized inexperienced operators and/or histopathologist reviewers. There was also a lack of consistency in the condition and methods of histopathological analyses. Variations in scoring systems, stains, section thickness, lighting conditions, and several other factors make it difficult to draw meaningful conclusions when comparing results from different studies (Wolf & Maack, 2017). To have comparable results across studies, a standardized set of operating procedures would be beneficial for environmental monitoring surveys.

Digital pathology is a powerful tool for image analysis that has already significantly transformed research in human health (Barisoni et al., 2020; Jones-Hall, 2021). Digital pathology refers to the analysis of scanned whole slide images on a computer, in contrast to traditional analyses under a microscope. Modern technology allows high-resolution scanning of whole histological slides that can be analyzed digitally. Digital analyses of whole slide images introduce possibilities of highly standardized histopathological evaluations. Incorporation of digital pathology into environmental monitoring studies using standardized bioindicator species or groups, such as bivalves and fish, can greatly improve the accuracy, reproducibility, and efficiency of the studies. Image analysis software allows scripting of workflows, which can be saved and distributed to other researchers. These scripts can then be used on compatible image sets by anyone who has access to the scripts and the software. Hart (2018) discussed and likened the evolution of digital pathology to the introduction of genomics, providing important details that can enhance the scientific advancement of such an approach. Although the benefits of digital pathology will be discussed within this review, method development will not be the focus, and readers are encouraged to reference Bankhead (2022) for more details.

Artificial intelligence (AI) has also been evolving at a rapid pace and may further assist in automated histopathological analyses. It has been used repeatedly in biomedicine, not only for descriptive histopathological evaluations, but also to diagnose patients (Ahmad et al., 2021; Rajpurkar et al., 2022; Sultan et al., 2020). However, the use of AI in histopathological analyses has been limited in environmental studies, potentially due to its perceived cost and complexity. Artificial intelligence can be applied at various levels of complexity and can aid in automated whole slide image analyses, which would reduce the time spent on analysis, as well as the cost and variance between studies. Furthermore, digital analysis would not only yield qualitative or semi-quantitative data that traditional scoring systems yield, but also fully quantitative data, which opens possibilities for more complex analyses.

This review aims to introduce readers to digital pathology and how it can be applied to environmental studies on various levels. This includes guidelines for sample preparation, potential sources of error, comparisons to traditional histopathological analyses, and examples of biomedical applications that could potentially be applied to environmental studies.

## From sampling to tissue slides

### Sample collection

**Figure 1.**
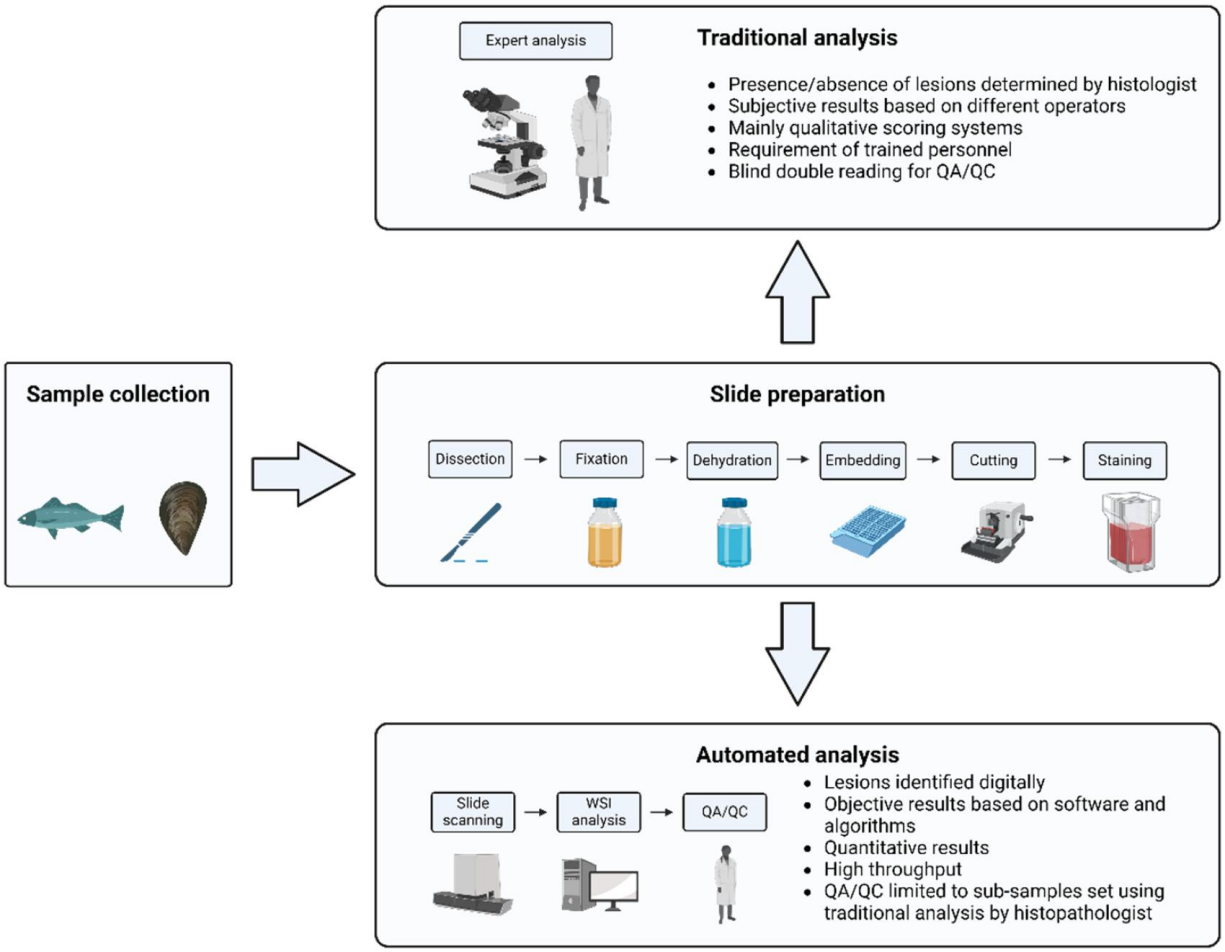
Workflow for histological analyses comparing traditional analysis to digital analyses using automated processes. WSI = whole slide image; QA/QC = quality assurance and quality control

The process of histological preparation can generally be divided into the following steps: dissection, fixation, dehydration, embedding, sectioning, and staining (Figure 1). Standardization of these steps is crucial for the production of quality data because even minute variations can result in false positives or negatives, especially for digital pathology applications. Standardization entails the utilization of techniques and practices commonly approved and used among scientists. Published guidelines, such as those provided by Bignell et al. (2012), Costa (2017), Howard (2004), and Kim et al. (2006), represent practices of which histologists should be mindful. Clear descriptions of correct sampling techniques are available in previously published materials (Howard, 2004), and in the online supplementary material Figure S1.

Lesions of interest should be kept in mind from the early stages of sample preparation. Each step of sample preparation could affect lesions differently and these possibilities must be considered to avoid false positives/negatives during analyses (da Silva & Villalba, 2004; Kindle et al., 2011; Wolf et al., 2015). Furthermore, different organs are composed of various tissue and cell types, which perform different physiological functions. As a result, they can be affected in different ways by xenobiotic compounds, and each tissue can present unique pathologies. Pollution-related lesions can be categorized in several ways and often co-occur with other pathologies and different metabolic processes. Therefore, the choice of targeted lesions should be made according to the goals of the study and their biological implications/meaning. Standardizing the set of histological lesions and their respective scoring systems used in environmental monitoring programs must be a priority to generate comparable data for large-scale assessments and multiple year comparisons.

### Dissection

Fresh tissues can be easily damaged so great care must be taken during dissections. For small organisms, it may be easiest to prepare cross-sections of the entire organism, rather than isolating specific organs, to prevent potential damage from additional handling and dissection. Sterile technique must be practiced to avoiding cross-contamination (e.g., avoid using the same instrument for cutting male gonads that is used to remove female gonads and between individuals). Guidelines for dissection are available for mussels (Lőw et al., 2016) and fish (Gupta & Mullins, 2010).

### Fixation, dehydration, clearing, and embedding

A variety of fixatives are used for histological preparation, none of them being a definitive standard for general use. An ideal fixative rapidly penetrates tissue to prevent postmortem degeneration, hardens tissue so it is not altered by histological processes, and protects tissue from shrinkage and other misleading structural changes. However, artifacts generated by fixatives when used on certain organs have been reported (Speare & Ferguson, 1989). Therefore, it is important to consider the advantages and disadvantages of certain fixatives (summarized in Howard, 2004). The time of permanence for fixatives is in most cases different in different studies/laboratories and can often be driven by logistical constraints. Standardization of fixation is also important for good quality results, and a fixing time between 24 hr and 48 hr is typical but can vary depending on application. This variance must be kept in mind if digital pathology is applied, because minute differences such as artifacts and different preparation methodologies can greatly affect automated analyses.

Dehydration and clearing, as well as paraffin wax infiltration, are usually carried out using automated tissue processors whereas embedding is typically done by hand. The use of automated tissue processors is encouraged for optimal wax infiltration and reproducibility, which directly affects all downstream histopathology-based methods. Waxes such as paraffin are commonly used for embedding tissue to maintain structural integrity while also allowing the application of various staining techniques. It is also crucial to assess the best method for embedding, because paraffin may not always be the best option. For instance, Melo et al. (2007) found that using plastic resin (glycol methacrylate) improved the ability to image the interior structure of copepods compared with the classic paraffin. More details on histological preparations are available in a publication by Al-Sabawy et al. (2021).

### Microtomy/sectioning

Sectioning is performed manually and there are several points where variance between samples can be introduced. In marine organisms living in direct contact with sediments or other surfaces, challenges like the occasional presence of sand granules or residual byssus, particularly in bivalves, may be encountered during sectioning and may prevent suitable sections from being obtained. These objects must be removed carefully from the block face to avoid damage to the surrounding tissues (Howard, 2004). Unfortunately, artifacts from folding or tearing tissues are frequent and, if not identified properly, may impair the dataset. It is also possible to exclude these regions from histological evaluations, although doing so could lead to bias in the assessment.

Section thickness should also be standardized between all samples, generally falling between 3 and 6 µm. Heterogeneity in section thickness is generally unavoidable and primarily occurs through the warming and expanding of the histology block during sectioning following removal from a cold plate. However, there are several factors that can affect the heterogeneity of sections that should be kept in mind. For example, the speed at which cutting/sectioning occurs should be as fast as possible without affecting the integrity of the cut. Slow cutting can increase the expansion of the block and, therefore, increase heterogeneity. Hardware issues can also result in heterogeneity between samples. A dull blade can easily introduce artifacts that may compromise the entire slide. More details on sectioning are available in Al-Sabawy et al. (2021).

### Slide staining

Haematoxylin and eosin stain (H&E) is a general-purpose dye for tissues fixed in formalin, where basophilic substances (nuclei) are stained in blue and acidophilic substances (cytoplasm, extracellular matrix) appear pink (Howard, 2004). It is commonly used in environmental monitoring (Hylland et al., 2008; Pampanin et al., 2019) and can be used for detecting a large variety of lesions. However, some lesions require further differentiation, in which case additional staining methods may be needed. For example, Alcian blue can be used to detect acid mucopolysaccharides, which can be useful in detecting abundant mucous secretion in gills (Howard, 2004). Fontana Masson, Nile blue, and Schmorl’s stain can be used to target lipofuscin and other pigments. Mallory’s trichrome and Van Gieson's stain are commonly used to detect collagen and connective tissue, which can aid in detection of fibrosis in fish liver (Auffret, 1988). Although it is not a specialized stain for this lesion, May–Grünwald Giemsa is commonly used to detect neoplastic cells (Burioli et al., 2019; Gorbi et al., 2012) whereas Oil Red O staining targets lipids (Marigómez et al., 2013). Table 1 summarizes commonly used stains, as well as targeted cellular components, target organs, and associated lesions, which could be adopted in various environmental monitoring studies according to their purpose.

**Table 1.** Staining methods suggested for environmental monitoring (Pampanin et al., 2019).

Staining technique	Targeted cell component	Addressed lesion	Target organ	References
Alcian blue	Mucopolysaccharides	Abundant mucous secretion	Gill	Howard (2004)
Fontana Masson	Lipofuscin	Lipofuscin accumulation/ melanomacrophage aggregates (MMA)	Gonad	Akaishi et al. (2007); Smolarz et al. (2017)
Mallory’s trichrome	Collagen	Fibrosis	Gonad	Auffret (1988); Gilek et al. (1992)
May–Grünwald Giemsa	Tumorous cell	Neoplasia	Organism	Cappello et al. (2013); Grizel et al. (2003)
Nile blue	Lipofuscin	Lipofuscin accumulation/MMA	Digestive gland	Auffret (1988)
Oil Red O	Lipid	Adipogranular tissue (ADG)/neutral lipid accumulation	Gonad/digestive gland	Brooks et al. (2010); Seed (1969); Schuster et al. (2021); Sundt et al. (2007)
Periodic Acid Schiff	Mucopolysaccharides	Abundant mucous secretion	Gills	Huertas & Bellwood (2020); Lichtenfels et al. (1996)
Schmorl’s stain	Lipofuscin	Lipofuscin accumulation/MMA	Digestive gland	Aarab et al. (2008); Battistini et al. (2020); Papo et al. (2014); Schuster et al. (2021)
Sudan black B	Lipofuscin	Lipofuscin accumulation/MMA	Gills	Smolarz et al. (2017)
TUNEL assay	Apoptotic cells	Apoptosis	Gonad	Marigómez et al. (2006)
Toluidine blue	Haemocytes	Haemocytes infiltration	Digestive gland	Howard (2004)
Van Gieson's stain	Collagen	Fibrosis	Organism	Genten et al. (2009)

Slide staining can easily result in variance depending on a number of factors, such as the previously discussed section thickness. The two most common sources of variance originate from the staining duration and the preparation of the H&E solutions (e.g., eosin acidity). Although staining duration can easily be standardized, variance from preparation of staining solutions can be more difficult to reduce, because there are several different vendors whose products all look slightly different from one another. Prezja et al. (2022) analyzed the variance of H&E staining between laboratories and include excellent figures that exemplify the heterogeneity in appearance of the same tissue stained by the same method but in different laboratories. However, they have also concluded that tissue type can also affect the variability of H&E stain appearance, which should also be considered. Some groups have utilized color standardization and normalization algorithms to homogenize the appearance of digitized H&E-stained slides (Janowczyk et al., 2017; Khan et al., 2012; Monaco et al., 2012), which provides the option for postprocessing standardization of slides that have already been stained. Specific information regarding histological staining is available in the bibliography (Alturkistani et al., 2016; Javaeed et al., 2021).

## Slide scanning and whole slide imaging

Technological advancements have made digital analysis of whole slide images easy to access and an attractive supplement to traditional microscopy analysis. Whole slide image analysis requires an additional step of scanning the microscope slide to create a single high-resolution digital file. This is commonly achieved by capturing many small high-resolution image tiles or strips, which are then assembled to create a full pyramidal image of the histological slide. These images contain complex data and are captured at a high resolution, which allows the detection of cellular or even subcellular structures (Pantanowitz et al., 2011). This enables the detection or quantification of structures and lesions with greater accuracy and precision compared to traditional methods (Cervellione et al., 2017).

However, imaging also adds an additional potential source of error for histopathological analyses. Subtle differences in lighting can easily be adjusted in traditional microscopy analyses but become a more onerous and time-consuming task for scanners, because the operator may not notice the lighting issue until after the scans have been complete. Most scanners can use a calibration slide to reduce this issue. Although lighting issues should be minimized as much as possible before scanning, they can also be mitigated in postprocessing by converting images from the red, green,blue (RGB) color space to the hue, saturation, intensity space (Doyle et al., 2010). This conversion limits differences in lighting between images to a single channel (intensity), as opposed to all three channels if using RGB, reducing heterogeneity between images. Some regions on the slide may also appear out of focus, which can arise from variations in tissue thickness, errors during mounting, or air bubbles under coverslips. Artificial intelligence tools can limit imaging errors by identifying areas containing artifacts (e.g., out-of-focus, tissue folds, air bubbles). Such approaches have already been proposed in clinical practice and consist of artifact segmentation networks to improve algorithm robustness (Smit et al., 2021) or diagnostic models (Schömig-Markiefka et al., 2021). Misalignments during stitching can also lead to jarring features that may interfere with lesion identification. Imaging conditions should be optimized using low magnification before a large number of slides can be scanned to minimize these errors. There are open-source quality control tools, such as HistoQC (Janowczyk et al., 2019), which can automatically perform quality control on whole slide images. For example, HistoQC can scan for artifacts, blurriness, heterogeneity in lighting and staining, edge detection, and several other potentially confounding factors. Although there are many variables to consider, once whole slide image scanning has been optimized, the benefits far outweigh the potential sources of error of digital pathology.

## Lesion evaluation

### Lesion identification

**Figure 2.**
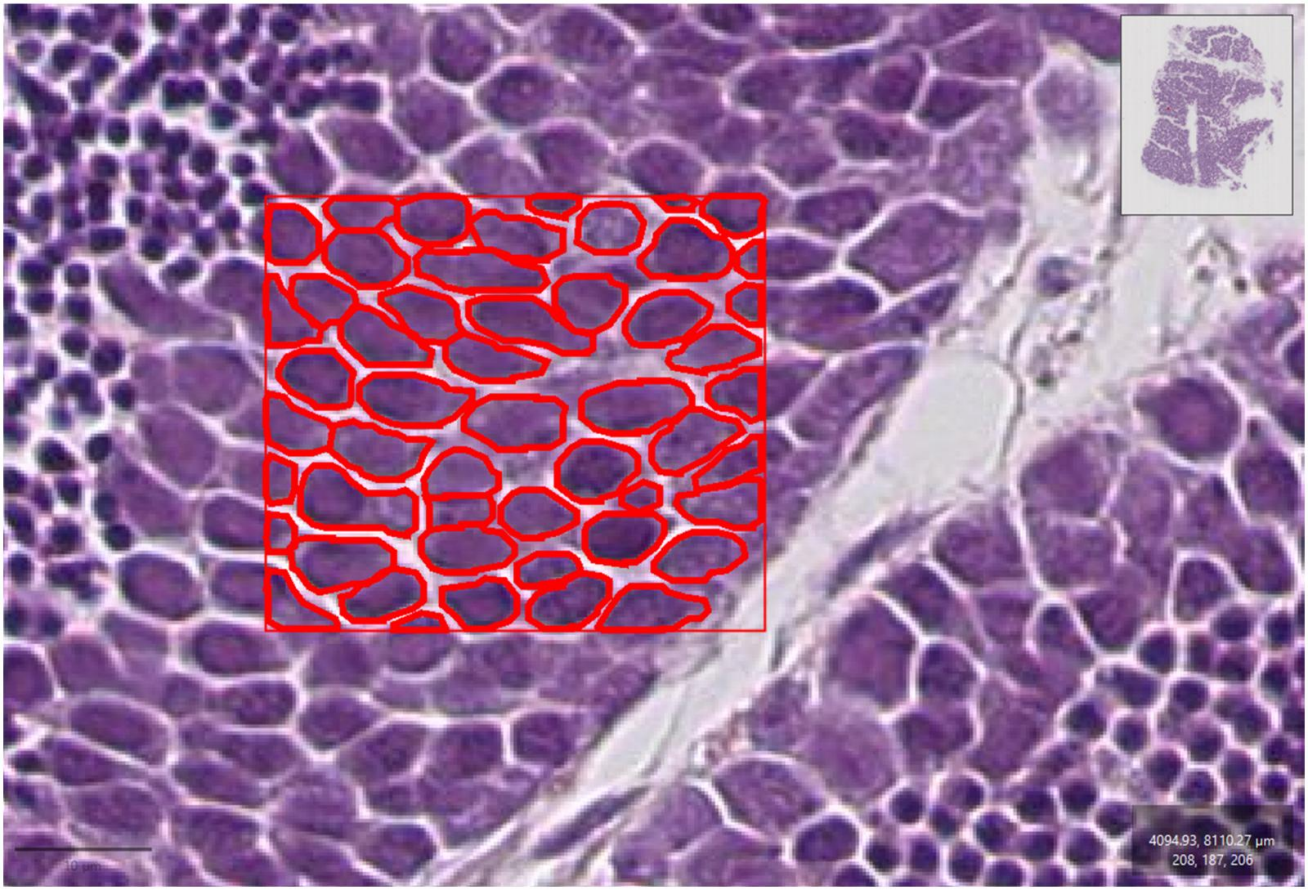
Example of cellpose being used to automatically detect and outline cells within a selected area of a whole slide image. Images were generated and analysis was conducted in QuPath.

Digital pathology has become an accessible tool for a wide range of applications, including environmental research (Barisoni et al., 2020; Sun et al., 2023; Szwedo, 2020). Powerful open-source software, such as ImageJ and QuPath, are available to download without user fees. Through the use of built-in functions and the support of plugins and scripting, lesion identification and detection can potentially be completed in a simple and replicable manner. Although there are few published automated protocols for lesion detection, there are published tools for similar functions. For example, the SlideToolkit utilizes a series of scripts to automate the workflow of detecting and quantifying 3,3'-diaminobenzidine (DAB)-stained tissues in whole slide images (Nelissen et al., 2014). An intraobserver intraclass correlation coefficient of 0.99 was observed in its application on 303 whole slide images, indicating high accuracy and reproducibility. Histolab is a python library for digital pathology preprocessing with automated testing (Marcolini et al., 2022). It is designed to be easily integrated into any computational pipeline, can automatically detect early signs of design flaws, and is capable of carrying out versatile tasks such as tissue detection, slide tilting, and removal of artifacts. Although these tools have mainly been applied in biomedical studies, they could be used to develop a digital processing framework for sharable standard operating procedures (SOPs) for ecological studies. This SOP can then be utilized by anyone with access to a computer that meets the hardware standards of the protocol. Although this type of SOP can be performed manually, the main benefit is the potential for automation. Automated image analysis software can perform repetitive tasks with great objectivity and consistency and has already been used in several cases (Clunie et al., 2018; Foley, 2009). Modern image analysis software allows the generation of executable scripts entirely through the user interface in a manner similar to macros. In some circumstances, complex analyses may require addition of custom code to the scripts, but simple and routine analyses may be automated even by users who do not know how to code. It should be noted that there exist many extensions for open-source software, which should always be searched for before investing time into writing novel code for a complex task. For example, the cellpose extension for QuPath allows the accurate quantification of number of cells, nuclei, and their areas within whole slide images (Bankhead et al., 2017; Cutler et al., 2022; Pachitariu & Stringer, 2022; Stringer et al., 2021). An example of cellpose being utilized to automatically detect and quantify cells is shown in Figure 2. Extensions like cellpose may come with prewritten code that just must be copy/pasted into the software to execute. This is usually available without user fees, but authors of the extensions may request citations for use.

Overall, digital analysis introduces the possibility for fully quantitative data, whereas only qualitative and semiquantitative data is typically used for traditional analyses. Manual microscopic analyses quantify severity using scoring systems, which is a discrete variable. Whole slide image analyses can provide exact measurements, a continuous variable, which can help refine health parameters with better accuracy. Furthermore, the use of AI and machine learning, which are only applicable to digital pathology, could allow analyses that are on par or even more accurate than trained histopathologists (discussed in detail later in the AI/machine learning section).

### Scoring systems

Manual microscopic analysis is usually performed by a trained operator or a histopathologist, who examines each slide to detect histological lesions and assesses their severity. This introduces a point of variance between operators, because generic scoring systems applied to several pathologies have a greater chance to be poor representations of the actual severity of the pathology. For instance, a scoring system was developed by Bernet et al. (1999) to assess the degree of severity of different pathologies on a scale. Score values ranging from 0 to 6 were used, with 0 representing no presence of the pathology in the tissue, 2 for mild occurrences, 4 for moderate occurrences, and 6 for the most severe occurrences. This type of scoring system is well adapted to diffuse lesions, such as abundant mucous secretion, but multifocal lesions, such as melanomacrophage aggregates, are difficult to assess because they often cover a smaller area of the tissue compared to diffuse lesions. Therefore, the degree of severity of punctual lesions will be constrained in the lower value of this scoring system, potentially leading to underestimations in severity assessments. Another commonly used scoring system is the histopathological condition index, adapted from Bernet et al. (1999). The index is estimated according to the concepts of differential biological significance of each alteration (weight) and its degree of dissemination (score). The weight is a value that ranges between 1 and 3, with 3 being the maximum severity value. The histopathological condition indices are estimated according to the following formula:


$$I_{h}=\frac{\sum_{1}^{j}w_{j}a_{jh}}{\sum_{1}^{j}M_{j}}$$

where I_h_ is the histopathological condition index for the individual h; w_j_ is the weight of the j^th^ histopathological alteration; a_jh_ is the score attributed to the h^th^ individual for the j^th^ alteration, and M_j_ is the maximum attributable value for the j^th^ alteration (weight × maximum score). The equation’s denominator normalizes I_h_ to a value between 0 and 1, thus permitting comparisons between distinct situations such as different organs (Costa et al 2013). This method has the advantage of being more precise and more comprehensive; however, it remains based on qualitative data, which do not fully express the severity or spread of a lesion. It is also difficult to statistically compare results from studies that used different scoring systems, especially if the variable types are different (ex. continuous vs. discrete). The issues of different scoring systems, combined with the inherent operator variance of scoring systems in general, stress the need for fully quantitative data.

### Method validation

Finally, quality control by an expert is required to validate the automated analysis. Even in studies with human histopathologists, variance and inconsistencies can be found between studies that set out to complete the same objective. For example, Wolf et al. (2014) describes a pathology working group evaluating three studies that observed the toxic effects of diclofenac in trout. They were able to identify several diagnostic inconsistencies and found that the no observed effects concentrations varied widely between the studies. This type of expert quality control is especially important for automated methods, particularly when batch-processing large numbers of whole slide images. It is recommended that an expert manually reviews a percentage of the processed samples to assure that the analyses are being conducted as expected. Although there is no set number for what percentage of slides to manually review, it largely depends on how many total slides were analyzed. For example, 20% can be easily reviewed for 100 slides, but if 100,000 slides were used, then even 5% would be an onerous task. For laboratories without access to a trained histopathologist, it is possible to outsource the analysis of a percentage of slides to a histopathology company to confirm the validity of the automated analyses. Future approaches can also combine the use of AI and manual control for quality control purposes and assessments of artifacts (Janowczyk et al., 2019; Smit et al., 2021).

### Statistical evaluation

Traditionally, a number of statistical tests have been used for lesions using scoring systems, such as the Mann-Whitney U, weighted kappa, chi-square, and Fisher’s exact test, depending on the type of data generated and desired analysis (Bedossa et al., 2012; Rozario & Ramakrishna, 2003; Treuting & Boyd, 2019). However, inappropriate use of parametric tests has been recognized as a frequent occurrence in scientific literature (Shott, 2011; Strasak et al., 2007), particularly for ordinal data such as scoring systems. As a result, the International Harmonization of Nomenclature and Diagnostic criteria has discouraged the use of statistical tests for pathologists using scoring systems due to the nonlinear grading scale and semiquantitative nature of the data (Mann et al., 2012). Instead, they recommend descriptive morphologic interpretations of a dose-response effect. Digital pathology allows the generation of fully quantitative data that can be analyzed with more statistically robust tests, including parametric analyses such as analysis of variance. This can reduce the subjective conclusions drawn from descriptive morphologic interpretations, which can vary between individuals, and open doors for standardized and reproducible analyses of histological data.

## AI/machine learning

Artificial intelligence is a broad term encompassing terms such as machine learning, neural networks, and deep learning. Although the terms are closely associated, they should not be used interchangeably.

Machine learning is a subset of AI that “learns” from training images and predicts the contents of new images. Although machine learning encompasses multiple types of learning models, it is sometimes synonymously referred to as supervised learning, which requires annotated training images (Choi et al., 2020). Machine learning models utilize information from annotations to classify lesions within new images or, in other words, a human operator tells a machine what features they are looking for, then the machine predicts where the features are within new images. Human intervention is required before the analysis (annotations) and after, where a human operator analyzes the results and fixes the annotations or features of analysis for better results. This trial-and-error approach is a relatively simple AI-based analysis and typically does not take long compared with other types of learning models. For more information regarding supervised methods, please refer to Rashidi et al. (2019).

Deep learning is a subtype of machine learning that does not always require annotated images. This is a more resource-intensive approach, which often needs powerful computer hardware and a larger number of training images compared with basic machine learning, often in the order of several thousand. Deep learning utilizes a layered set of algorithms known as neural networks, which are designed to mimic the human brain (Choi et al., 2020). Unlike supervised machine learning, deep learning requires little human intervention. In addition to not requiring annotated training images, some models are even self-correcting (Du et al., 2021; Leino et al., 2022; Pan et al., 2019). Although deep learning is more resource intensive than machine learning, it often outperforms basic machine learning models and traditional data analyses (Janiesch et al., 2021).

Artificial intelligence-based methods can potentially analyze images on a deeper level, utilizing and providing information that may have been overlooked by even experienced histopathologists. Significant strides have already been made in medical applications. Artificial intelligence-assisted analyses of histological slides have not only yielded accurate diagnoses, but have also suggested treatment responses, charted cancer outcomes, and even predicted genetic alterations and gene expression of specific patients (Shmatko et al., 2022). Although these AI-assisted analyses can be powerful, they are not completely replacing human operators and, instead, are being used for prescreening to reduce the workload of histopathologists (Cifci et al., 2022). Kalra et al. (2020) reported that an automated AI-based diagnostic tool, when fed 30,000 high-resolution digitized slides, was 93%–100% accurate in diagnosing 32 different cancer subtypes. Another study found that diagnoses of breast cancer by detection of micrometastases was 91.2% accurate by AI-assisted methods compared with the 83.3% accuracy by a pathologist alone (Försch et al., 2021). Esteva et al. (2017) trained a convolutional neural network with 129,450 clinical images, then compared its diagnoses of cancerous lesions within 1,942 images to 21 board-certified dermatologists. The overall diagnostic accuracy by the neural network was 55.4%, which was on par with manual diagnoses by board-certified dermatologists who scored 54.2% on average. Similar results were observed with neural networks trained to detect lymph node metastases in women with breast cancer (Bejnordi et al., 2017), where the deep learning algorithm showed greater discrimination (area under the curve = 0.994) compared with the highest scoring pathologist (area under the curve = 0.884). Furthermore, Mobadersany et al. (2018) demonstrated that a deep learning algorithm was able to accurately predict the survival of patients by assessing brain tumors in whole slide images, the predicted accuracy (c index = 0.754) being higher than manual histologic grading (c index = 0.745), although not statistically significant. Although many of these studies used complex neural networks that are often out of reach for ecological research due to costs and resource availability, Sailer et al. (2021) observed that, compared with convolutional neural networks with complex architecture (e.g., VGG16, ResNet50), simpler networks that were trained from scratch with minimal preprocessing and without much cost (e.g., no graphics processing unit utilization) yielded robust results and reportedly had a better computational cost/performance tradeoff, although exact cost/performance values were not published. Du et al. (2016) reported that a small network (based on the number of nodes) can achieve greater predictive performance than traditional machine learning approaches, with the deep learning approach (deep belief network) scoring an accuracy of 86.0%, whereas the two traditional approaches (support vector machine and gradient boosting model) scored 84.0% and 85.5%, respectively. Additional deep learning use cases were reported by Janowczyk and Madabhushi (2016).

Despite the progress in human health research, applications of AI-based methods, or even non-AI automated digital pathology methods are sparse in environmental studies. Szwedo (2020) developed an automated method for determining the sex and spawning stage of blue mussels (*Mytilus edulis*), using digital whole slide images of their gonads, which had a success rate of over 97%. Sun et al. (2023) incorporated a machine learning-based digital pathology analysis to establish a correlation between lipid profiles and whole organism effects in earthworms (*Eisenia fetida*) exposed to molybdenum disulfide nanosheets. At the time of writing this review, these were the only two published examples to our knowledge that used automated histological analyses in ecological studies. If a fraction of these machine-assisted tools utilized in the medical field were applied to environmental studies, they could greatly reduce operator variance, allow nonexpert histopathologists to run analyses, and save both time and money for environmental surveys.

**Figure 3.**
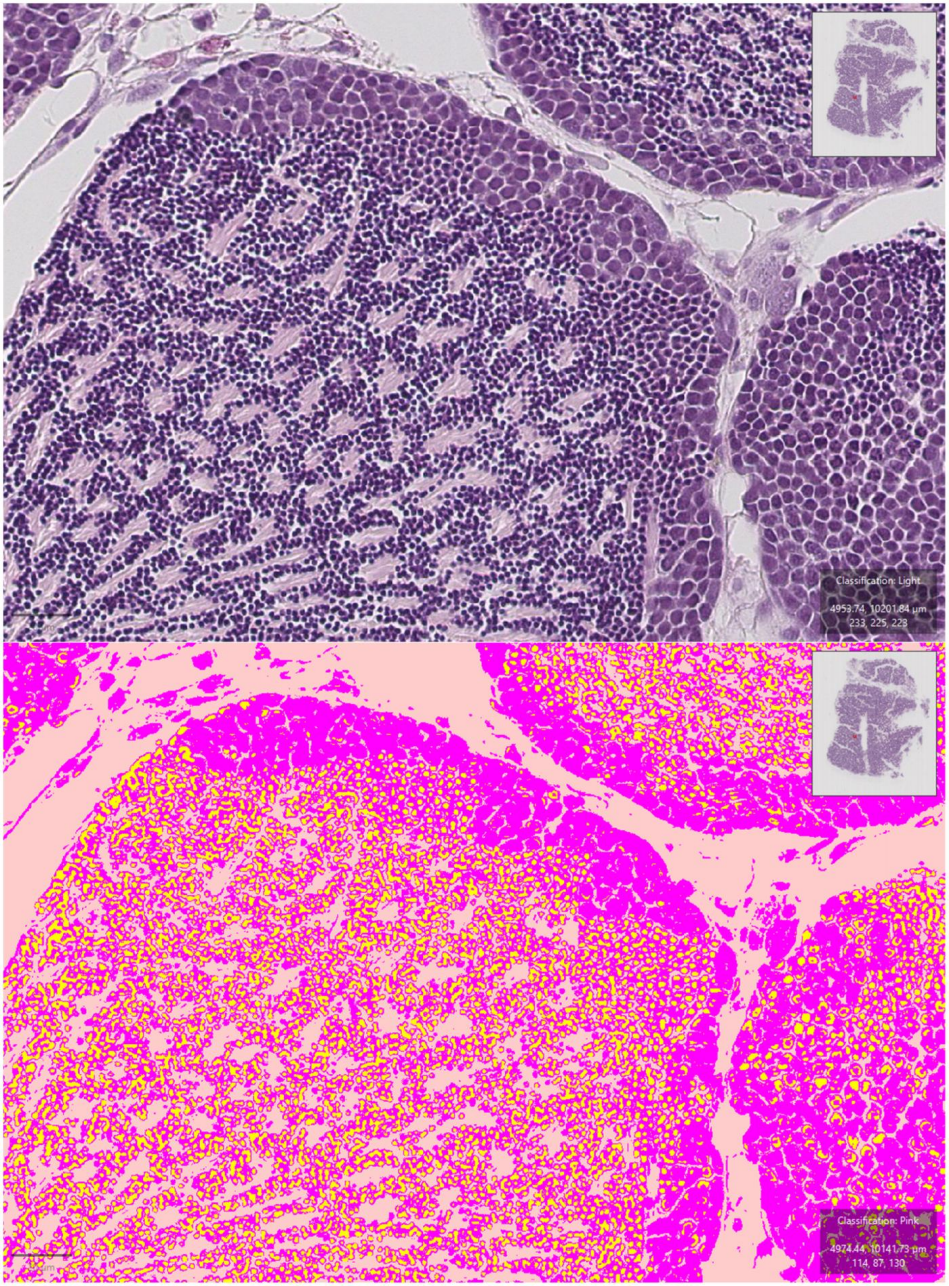
Example of a pixel classifier automatically classifying different features in a whole slide image using annotated training sets. Images were generated and analysis was conducted in QuPath.

**Figure 4.**
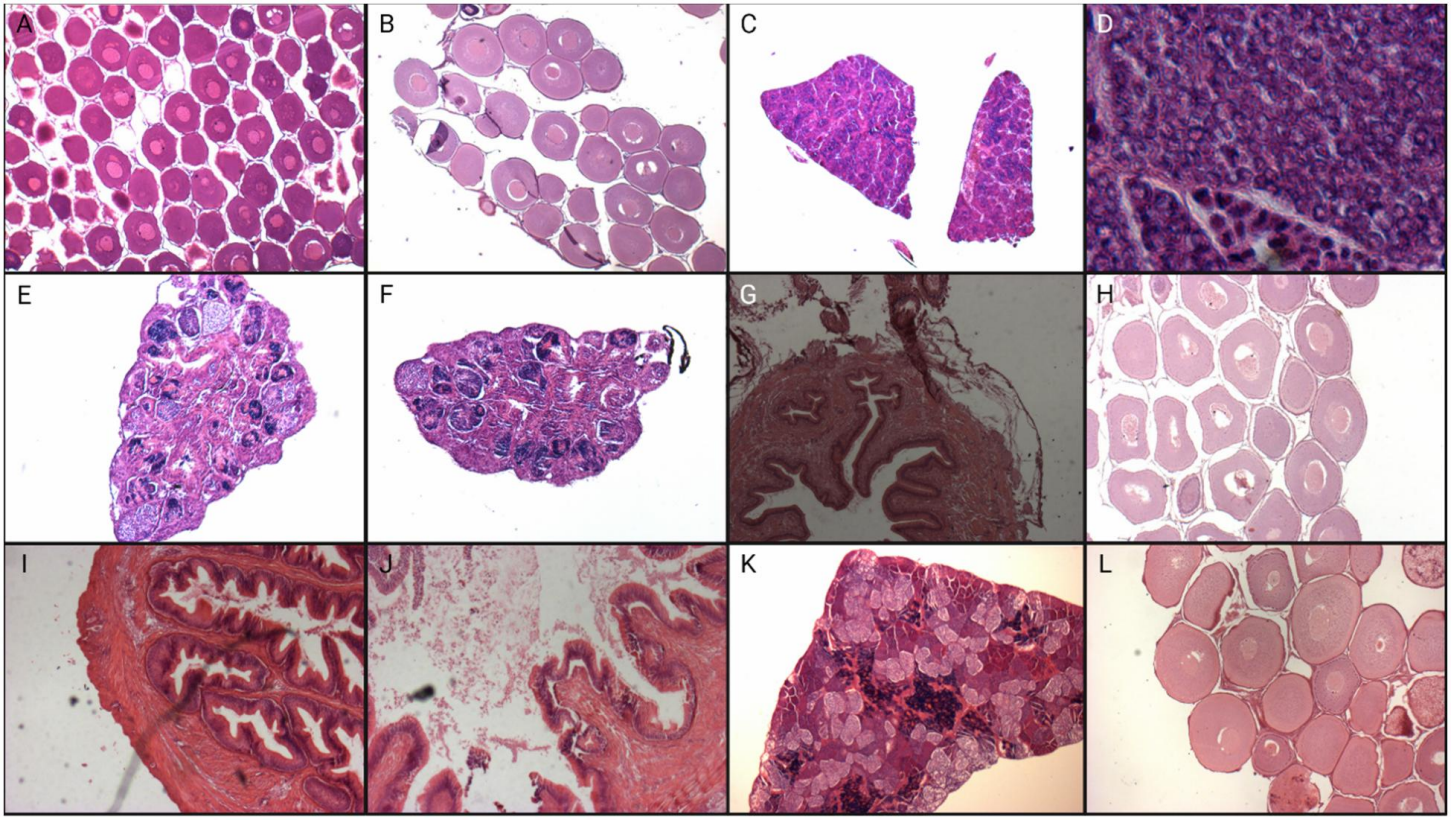
Commonly observed examples of artefacts found in histological analyses. (A) Blade mark and dehydrated; (B) folding; (C) out of focus; (D) out of focus/thick tissue section; (E) poor fixation; (F) poor fixation/sectioning; (G) poor sectioning and lighting; (H) poor staining and dehydrated; (I) slide debris; (J) staining debris; (K) uneven lighting; and (L) white balance off, artifact in view.

Although AI-based methods have great potential, human and machine-based histopathological analyses also have challenges. Traditional identification of lesions relies entirely on the human decisions of the operator. The operator looks for certain key features and determines what lesion they are looking at. This could, at times, lead to subjectivity. If certain key features are weak, one operator may choose to discount the lesion as background, whereas another operator may determine it to be significant. Even if the same operator finds another lesion with the same weak features, they may choose to deem it significant the second time around. Machine-based detection can vary in its methodologies. Machine-based methods can make human-like decisions if scripted to make decisions based on certain if/then statements, which is an automated procedure but not necessarily an AI-based approach. They can also make decisions entirely based on pixel data and pattern-recognition if a classifier was trained with annotated images or if a neural network was fed a large dataset for nonannotated analysis. An example of a pixel classifier used to automatically classify different tissue types in a whole slide image is shown in Figure 3. This can help reduce subjectivity, which affects human analyses but, unlike humans, machines often fail to consider extraneous information like the presence of artifacts. As discussed earlier, artifacts are imperfections originating from errors during the preparation and scanning of a slide and are often observed during later stages of analyses. They may hinder the identification or quantification of lesions by automated methods because they can greatly vary in appearance. Therefore, it is important to keep track of any event that happens during sample collection and preparation. Commonly observed examples of artifacts are shown in Figure 4. Artificial intelligence-based methods are also difficult to quality control or validate because of the difference in analytical methods compared with humans. Humans typically find key features, such as lesions, from which they make conclusions. An artificial intelligence-based analysis, particularly deep learning, does not always utilize this approach and looks at all information available within the image to draw a conclusion. This makes it difficult to determine how the analyses are being conducted and if the conclusions are being drawn based on valid, physiologically relevant information. For this reason, a large sample size, often in the order of thousands to hundreds of thousands, is necessary, as well as manual review of a percentage of slides to validate the accuracy of the program. For the same reason, methods of automated analysis should balance the complexity of the task with the range of applicability. A supervised machine learning approach or simple algorithms can be fine-tuned and potentially utilized for a wide range of applications, whereas a deep learning analysis can be much more powerful but difficult to adjust, limiting its range of applications.

## Conclusions

The use of digital whole slide image analyses represents a paradigm shift in histopathology and can be used to better standardize histopathological analyses, particularly in environmental studies. By digitizing whole microscopic slides, a multitude of computerized analytical techniques are available for use. Digitization can streamline workflows, improve reproducibility, standardize lesion identification, and facilitate global collaboration. However, the most significant benefit in whole slide image analysis is automation. Automation can not only better standardize histopathological analyses but, with the use of algorithms and AI-based methods, standardize the identification, detection, and quantification of lesions as well. With established open-source image analysis software, such as ImageJ and QuPath, methods can be developed and available for use globally free of charge, which would further entice large-scale utilization without being limited by funds. Rapid development and application of digital pathology in human health research provides a template for its implementation for environmental studies. Furthermore, environmental studies commonly involve the measurement of specific biological endpoints, often in a few highly studied species, which requires far less complex analyses compared to medical applications. For highly standardized analyses like this, automated histological methods would be quite applicable and powerful. A standardized, open-access protocol for identification, detection, and quantification of lesions would revolutionize the field of histopathology and help create a wealth of robust, comparable knowledge for monitoring environmental health.

## Supplementary Material

vgae038_Supplementary_Data

## Data Availability

Data is available from the authors on request: philip.tanabe@noaa.gov.
