# Supplementary material for: Using digital pathology to standardize and automate histological evaluations of environmental samples
Source: Environ Toxicol Chem. 2025 Jan 6;44(2):306–17. doi: 10.1093/etojnl/vgae038 (PMC11816309; doi:10.1093/etojnl/vgae038)

**Supplemental Information**

*Philip Tanabe^1*^, Daniel Schlenk^2^, Kristy L. Forsgren^3^, Daniela M. Pampanin^4^*

*^1^National Oceanic and Atmospheric Administration, National Ocean Service, National Centers for Coastal Ocean Science, Charleston, SC*

*^2^Department of Environmental Sciences, University of California, Riverside, Riverside, CA*

*^3^Department of Biological Science, California State University, Fullerton, Fullerton, CA*

*^4^Department of Chemistry, Bioscience and Environmental Engineering, University of Stavanger, Stavanger, Norway*

*There are 7 pages with 1 Supplementary Figure and 3 publication licenses*

** Corresponding author:*

*Corresponding author: Philip Tanabe*

*Corresponding email: philip.tanabe@noaa.gov*

*Corresponding address: Hollings Marine Laboratory, National Oceanic and Atmospheric Administration, 331 Fort Johnson Rd, Charleston, SC 29412*

**Figures**


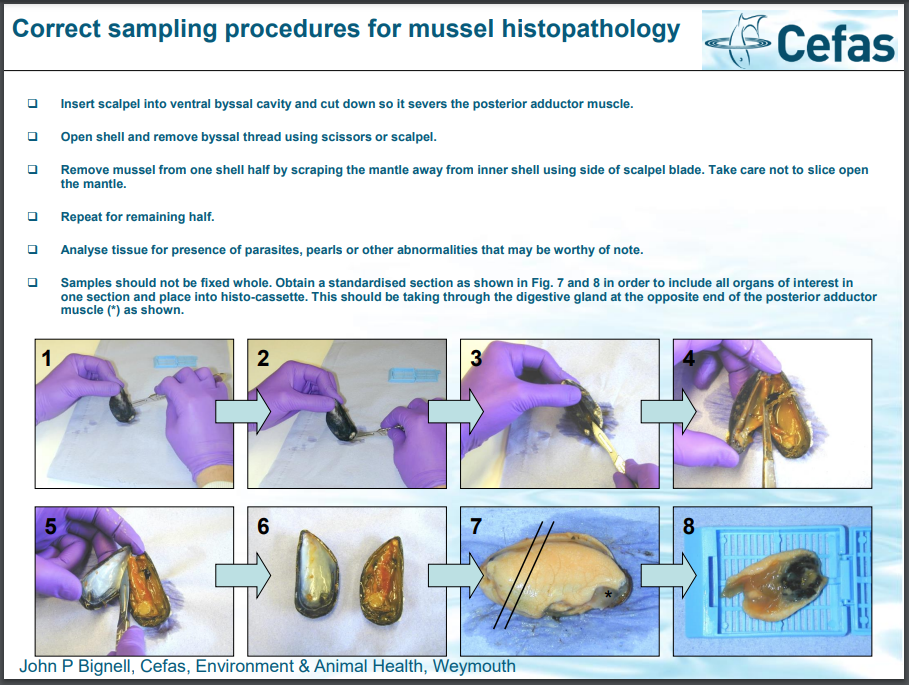


Figure S1. Correct sampling procedures for mussel histopathology.


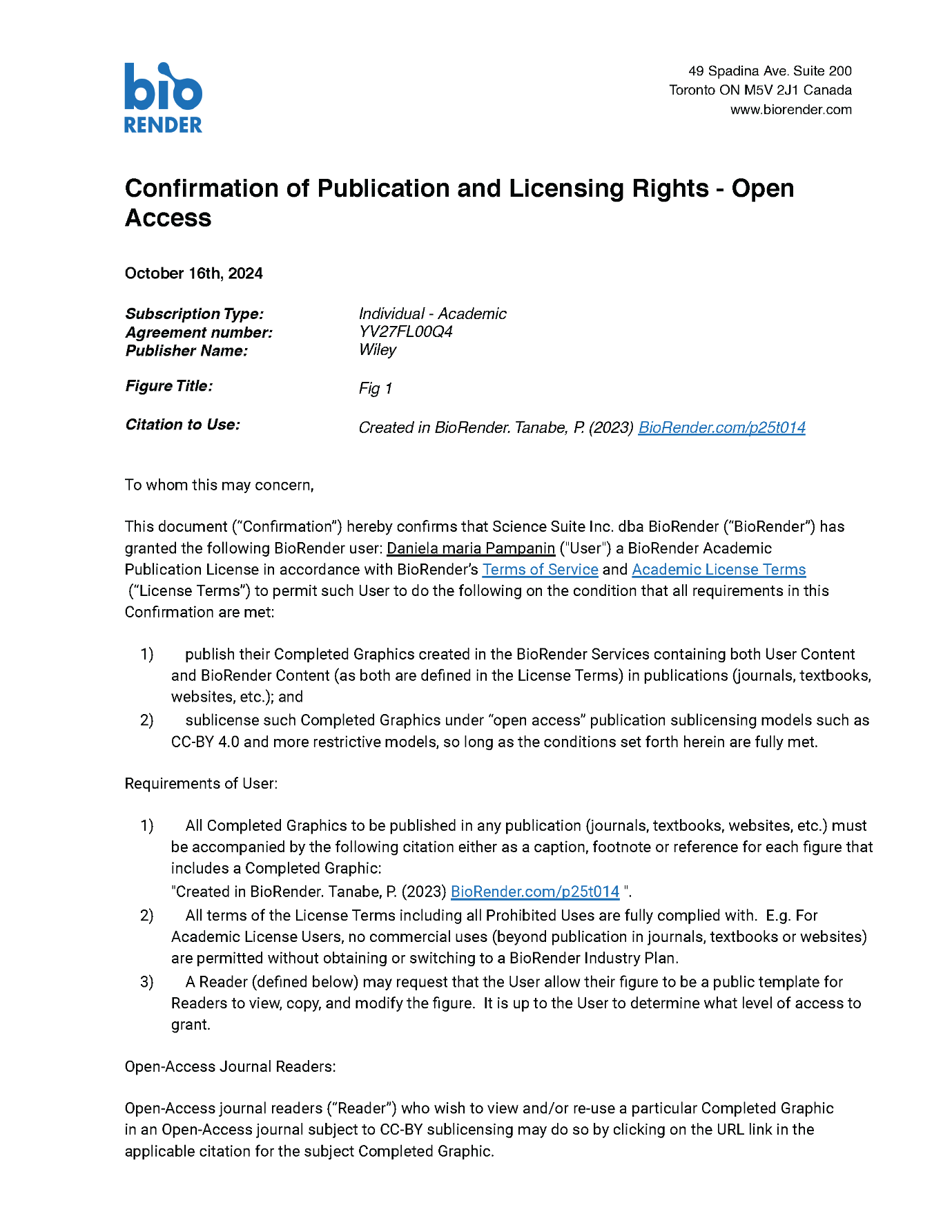
**Publication Licenses**

**
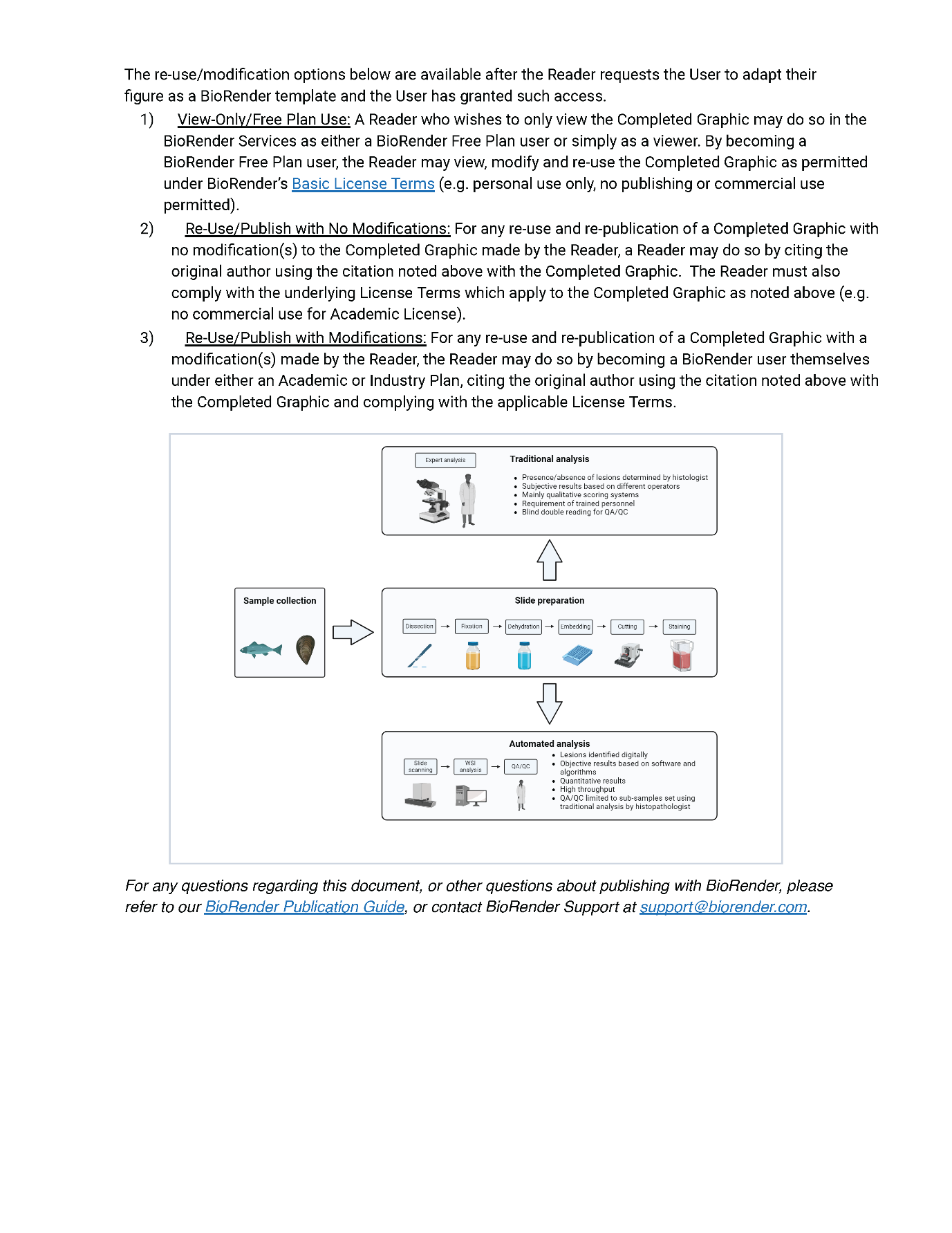
**

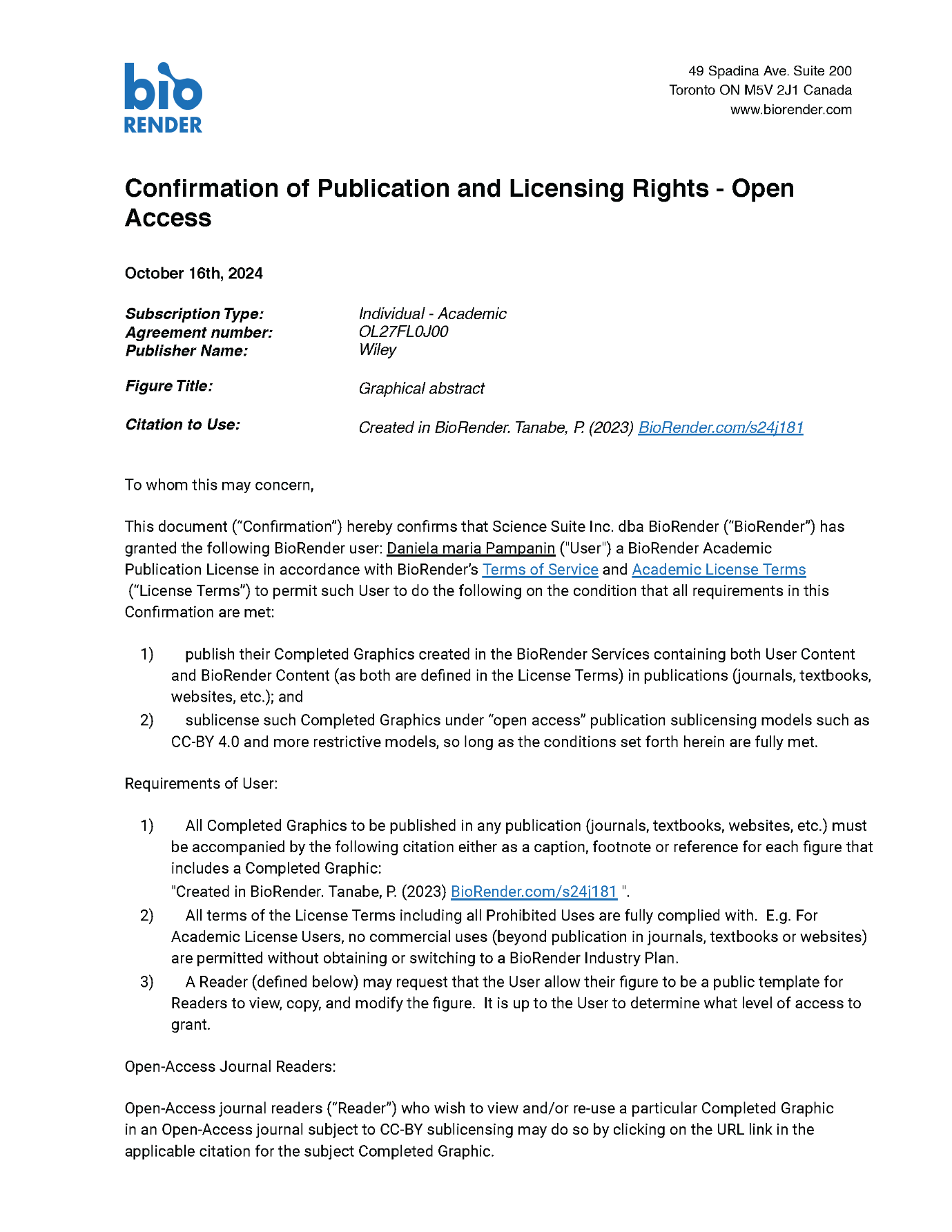


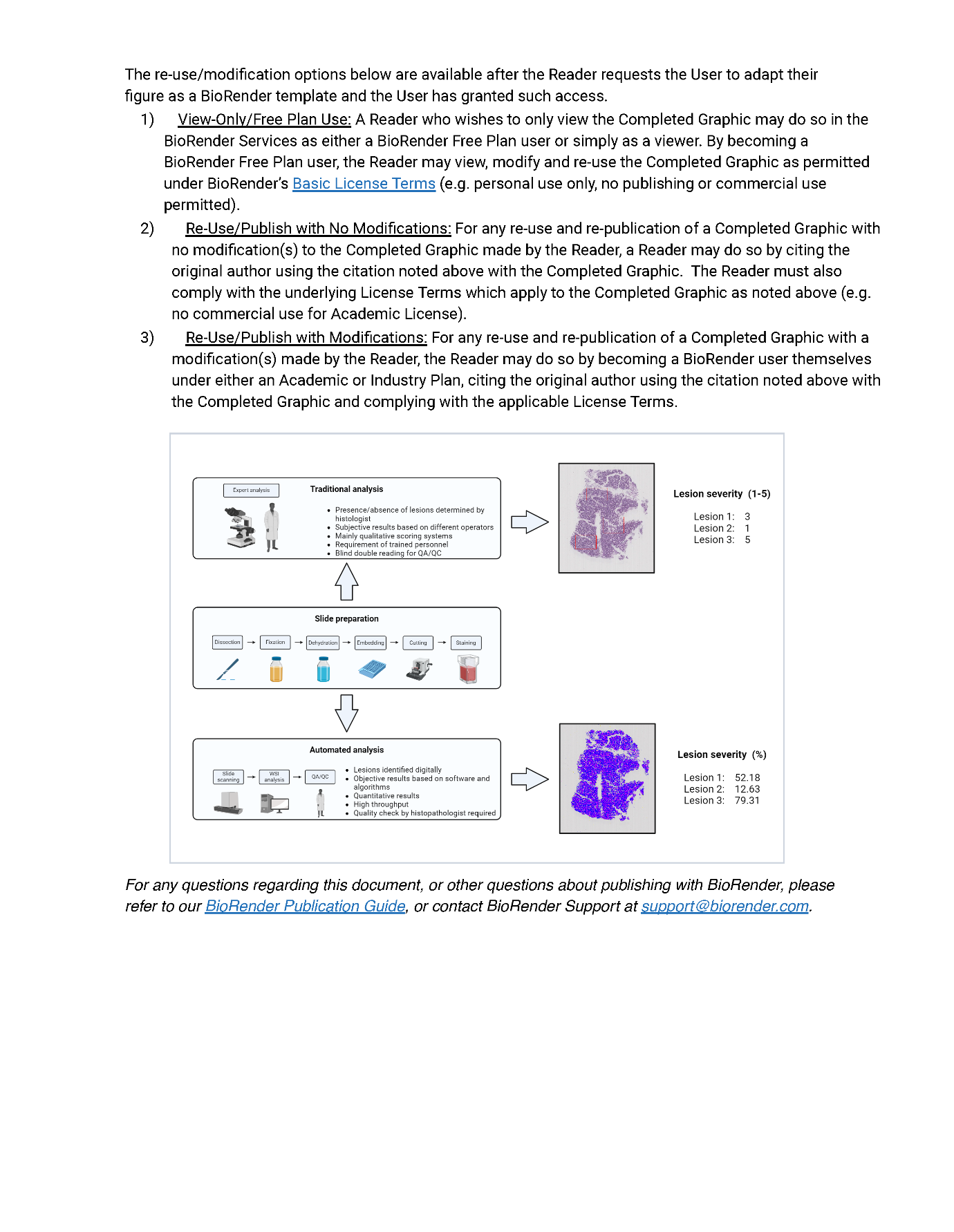

Supplement: vgae038_Supplementary_Data [file vgae038_supplementary_data.docx]
